# From evidence to policy: WHO’s COVID-19 infection prevention and control guideline development process

**DOI:** 10.4102/jphia.v16i2.1296

**Published:** 2025-06-30

**Authors:** Hannah M. Hamilton Hurwitz, Kathleen Dunn, Roger Chou, Nathan Ford, João Paulo Toledo, April Baller

**Affiliations:** 1 World Health Organization, Geneva, Switzerland; 2 Public Health Agency Canada, Ottawa, Canada; 3 Oregon Health and Science University, Portland, United States of America

## Introduction

The World Health Organization (WHO) follows a rigorous guideline development process to develop evidence-based policy and guide practice.^1^ Central to this process is a rigorous, reproducible and comprehensive evidence synthesis, which serves as the foundation for guideline development. At the start of the pandemic, the WHO’s infection prevention and control (IPC) emergency interim guidance for COVID-19 took an expedited approach,^2^ rapidly addressing emerging issues. Given the lack of direct evidence on IPC measure for COVID-19, guideline developers utilised indirect evidence from existing WHO guidelines on severe respiratory illness.^3,4^ The guideline later transitioned to using standard WHO guideline development methodology^1^ and using evidence directly addressing IPC measures or COVID-19, culminating in a comprehensive and consolidated guideline.^3,5^

Transitioning to the standard guideline development process involved identifying the core set of questions that required evidence synthesis; of which 32 research questions were identified by the guideline steering committee and guideline development group (GDG) and 17 systematic evidence reviews commissioned (steering community and GDG composition can be found in the acknowledgement section of the latest addition of the infection prevention and control in the context of COVID-19: A guideline).^3,4,5^ These reviews provided the basis for formulating recommendations through evidence synthesis and assessing the certainty of the evidence, with support of a methodologist. In addition, recognising the importance of contextual factors such as health and care workers’ values and preferences in developing recommendations, five qualitative evidence syntheses were performed to addresses these areas.^3^

## Systematic reviews

Key questions were reviewed simultaneously within a constrained timeframe. Given the substantial research demand, multiple groups were commissioned to conduct reviews. Some have already been published,^6,7,8^ while others are included in this supplement (Table 1 and Table 2). Additional systematic reviews were conducted independently and are not included here, but details are referenced in the guideline.^5^

**TABLE 1:** COVID-19 research questions available in this supplement.

Topic	Research question
Physical distancing	Should a physical distance of 1 metre be maintained to reduce and mitigate transmission of SARS-CoV-2?
Cleaning/disinfection	How does soap and water cleaning alone compare to bleach-based cleaners in eliminating SARS-CoV-2 infection?
	Does SARS-CoV-2 require differential cleaning in healthcare facility settings beyond standard environmental cleaning procedures?
	Should surfaces and materials in healthcare settings (where providing care to patients with COVID-19) be disinfected using a wiping method versus a spraying method?
	Is ultraviolet germicidal irradiation effective as an environmental (surface) cleaning measure in healthcare settings?
Mask use during physical activity	Should facemasks be worn during physical activity compared to not wearing a facemask in terms of protecting against COVID-19 transmission?
Testing	Should routine testing of asymptomatic health and care workers for COVID-19 surveillance be conducted?
	Should health and care workers be tested following a high-risk exposure to SARS-CoV-2?

SARS-CoV-2, severe acute respiratory syndrome coronavirus 2.

**TABLE 2:** Qualitative evidence synthesis questions available in this supplement.

Topic	Research question
Cleaning and disinfection	What are the perceptions of stakeholders and factors influencing the uptake of cleaning and disinfection interventions in community settings in the context of COVID-19?†
	What are the perceptions of stakeholders and factors influencing the uptake of cleaning and disinfection interventions in healthcare settings in the context of COVID-19?†
PPE and physical distancing	What are the perceptions and factors influencing the uptake of mask-wearing and physical distancing in the community settings in the context of COVID-19?
	What are the perceptions and factors influencing the uptake of mask-wearing and physical distancing in healthcare settings in the context of COVID-19?
Testing	What are the perceptions and factors influencing the uptake of diagnostic test interventions for infection prevention and control during the COVID-19 pandemic?

PPE, personal protective equipment.

† , These two questions have been combined into one article.

The reviews followed standardised systematic review methods, such as searching multiple databases, applying predefined inclusion and exclusion criteria, assessing risk-of-bias and determining evidence certainty using Grading of Recommendations, Assessment, Development and Evaluation (GRADE).^1^ Evidence from randomised controlled trials was often either non-existent or very limited; consequentially, the reviews were broadly inclusive of various study designs including non-randomised/non-comparative studies, observational, cohort, case-control and ecological studies. Bias and confounding were addressed as part of the GRADE process, often resulting in a judgement of low certainty evidence.

### Contextual issues addressed through qualitative evidence synthesis

Contextual issues related to the acceptability, feasibility, costs or resources, variability values and preferences and the potential impacts on equity, play a crucial role in the formulation of recommendations.^9,10^ Qualitative evidence synthesis of these contextual factors provides information that can be incorporated into evidence-to-decision frameworks and inform the development of recommendations, implementation considerations, and provide insights for the application of IPC measures.^9,10^ The use of qualitative evidence synthesis aids in identifying factors that may influence the implementation of health policies and interventions, and integrating perspectives and experiences from diverse stakeholders, including underrepresented groups,^9,10^ helping to ensure that guidelines are more acceptable and implementable.

### Assessing the evidence and recommendation development

World Health Organization convened a GDG to interpret the evidence, assess its quality, and formulate recommendations utilising the GRADE approach. The scarcity of high-quality data emphasises the importance of having a diverse representation of GDG members with expertise across various disciplines and represent the various regions around the world. The experience of this multidisciplinary group provided additional insight by leveraging their first-hand experiences to interpret the findings of the systematic review, to better inform the development of recommendations, including the strength assigned to the recommendations.

## Conclusion

While most of the details of how the COVID-19 IPC guideline was developed are available within the methodology and supplemental material associated with the guideline,^5^ the articles in this theme collection offer an in-depth, complementary perspective into the evidence available to the guideline panel at the time of writing. Amid the evolving landscape of the COVID-19 pandemic, balancing responsiveness with emerging IPC issues while reliably appraising the emerging evidence through systematic reviews proved challenging. These articles also mark a milestone in the guideline development process – a shift from the reliance on studies with indirect and/or very low certainty evidence and expert opinion to direct, robust evidence and standard methodologies.^3^ Furthermore, this themed collection highlights the research gaps and underscores the need for high-quality studies to establish a robust evidence base for future IPC guidelines in the context of respiratory infectious disease outbreaks/pandemics.^4^ Publishing these articles promotes transparency, provides a detailed record of the process, and serves as a reference for future adaptation. This will allow us to revisit and build upon the evidence as needed, should a future threat require the rapid development of guidelines, just as was done for COVID-19.^3,4^
